# LungMAP: The Molecular Atlas of Lung Development Program

**DOI:** 10.1152/ajplung.00139.2017

**Published:** 2017-08-10

**Authors:** Maryanne E. Ardini-Poleske, Robert F. Clark, Charles Ansong, James P. Carson, Richard A. Corley, Gail H. Deutsch, James S. Hagood, Naftali Kaminski, Thomas J. Mariani, Steven S. Potter, Gloria S. Pryhuber, David Warburton, Jeffrey A. Whitsett, Scott M. Palmer, Namasivayam Ambalavanan, The LungMAP Consortium

**Affiliations:** ^1^RTI International, Research Triangle Park, North Carolina;; ^2^Pacific Northwest National Laboratory, Richland, Washington;; ^3^Texas Advanced Computing Center, Austin, Texas;; ^4^University of Washington, Seattle, Washington;; ^5^University of California, San Diego, California;; ^6^Yale School of Medicine, New Haven, Connecticut;; ^7^University of Rochester Medical Center, Rochester, New York;; ^8^Cincinnati Children’s Hospital Medical Center, Cincinnati, Ohio;; ^9^Children’s Hospital of Los Angeles, Los Angeles, California;; ^10^Duke University School of Medicine, Durham, North Carolina; and; ^11^University of Alabama, Birmingham, Alabama

**Keywords:** lung development, web resource, lung imaging, lung omics, 3D imaging, single cell analysis

## Abstract

The National Heart, Lung, and Blood Institute is funding an effort to create a molecular atlas of the developing lung (LungMAP) to serve as a research resource and public education tool. The lung is a complex organ with lengthy development time driven by interactive gene networks and dynamic cross talk among multiple cell types to control and coordinate lineage specification, cell proliferation, differentiation, migration, morphogenesis, and injury repair. A better understanding of the processes that regulate lung development, particularly alveologenesis, will have a significant impact on survival rates for premature infants born with incomplete lung development and will facilitate lung injury repair and regeneration in adults. A consortium of four research centers, a data coordinating center, and a human tissue repository provides high-quality molecular data of developing human and mouse lungs. LungMAP includes mouse and human data for cross correlation of developmental processes across species. LungMAP is generating foundational data and analysis, creating a web portal for presentation of results and public sharing of data sets, establishing a repository of young human lung tissues obtained through organ donor organizations, and developing a comprehensive lung ontology that incorporates the latest findings of the consortium. The LungMAP website (www.lungmap.net) currently contains more than 6,000 high-resolution lung images and transcriptomic, proteomic, and lipidomic human and mouse data and provides scientific information to stimulate interest in research careers for young audiences. This paper presents a brief description of research conducted by the consortium, database, and portal development and upcoming features that will enhance the LungMAP experience for a community of users.

the lung is a complex organ with high cellular heterogeneity. Development and maintenance of the structure of the lung requires interactive gene networks and dynamic cross talk among multiple cell types to control and coordinate lineage specification, cell proliferation, differentiation, migration, morphogenesis, and injury repair (15, 18, 20, 24). Many key signaling molecules, genes, and pathways have been discovered, yet significant knowledge gaps still exist in understanding lung development from late fetal to perinatal stages and through early childhood. Little is known of human lung development at this critical period when the diverse lung cells go through terminal differentiation and maturation and when the gas exchange units (alveoli) form. Understanding alveologenesis has significant clinical relevance. Prematurely born infants, some as young as 23 wk in gestation, have incomplete lung maturation and morphogenesis that can lead to pulmonary dysfunction after birth, causing a disorder termed bronchopulmonary dysplasia (BPD) that may impair lung function in infancy and thereafter. Defects in lung morphogenesis before the alveolar stage contribute to respiratory functional deficiencies later in life (14, 19). How to promote lung growth, maturation, and alveologenesis in premature babies remains a clinical challenge. Among young infants with BPD and other childhood interstitial and diffuse lung diseases (ChILD), lung growth abnormalities make up the largest disease category associated with high morbidity and mortality (11). Knowledge about the molecular interactions among diverse lung cells, the genes that regulate their functions and behavior, and the molecular and physical interactions among the cells and their microenvironment during normal development will support novel approaches to advancing treatments for lung injury repair and regeneration. Molecular profiles of the diverse cell types in the lung (including epithelial, mesenchymal, neuronal, vascular, immune, and other non-endoderm-derived lineages), knowledge of the dynamics of three-dimensional (3D) cellular structure of the airways and alveoli, and an integrative open-access database to assimilate complex multidimensional data are needed to provide the foundation for a complete understanding of lung development. A molecular atlas of the human lung will be a critical platform for future research to inform basic mechanisms and treatments for childhood and adult lung diseases. Until we understand normal development, it is difficult to decipher what changes occur at the molecular level in both abnormal lung development (e.g., conditions such as congenital diaphragmatic hernia, congenital pulmonary airway malformation, or BPD) and adult lung disorders.

The LungMAP consortium (Fig. 1) was developed to generate detailed structural and molecular data regarding normal perinatal and postnatal lung development in the mouse and human. Data are openly available to the entire research community to advance this knowledge across research groups with an interest in understanding lung development. Key to the consortium was the establishment of centers of excellence in imaging and omics technologies, supporting tissue procurement and a data coordinating center.

**Fig. 1.**
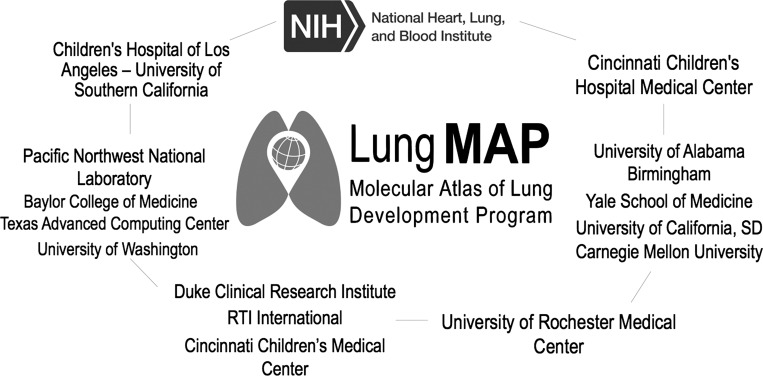
The LungMAP Consortium. Organizations funded by the National Heart, Lung, and Blood Institute for the Molecular Atlas of Lung Development Program. NIH, National Institutes of Health.

To date, LungMAP funds and efforts have contributed to the generation of data and tools used in 42 published manuscripts as well as numerous abstracts and posters at scientific conferences. Six of the journal articles reflect collaborative research by two or more LungMAP research centers (for a full list see https://lungmap.net/resources/publications/). Nearly all publications explore the transcriptomics, proteomics, or epigenetics of lung development. Three articles are reviews of existing published work, and four articles focus solely on bioinformatics and systems analysis work that has been accomplished by the LungMAP consortium. Disease conditions are included in 22 of the publications, which are focused on bronchopulmonary dysplasia and other complications of prematurity and idiopathic pulmonary fibrosis. Published work results include 14 that are specific to mouse experiments, 16 involving human studies, and eight that work with both mouse and human data. Although some human data is now offered on the LungMAP website, the abundance of human data that support the many published papers will be available for public sharing in early Fall of 2017.

## LungMAP: Who We Are

**Fig. 2.**
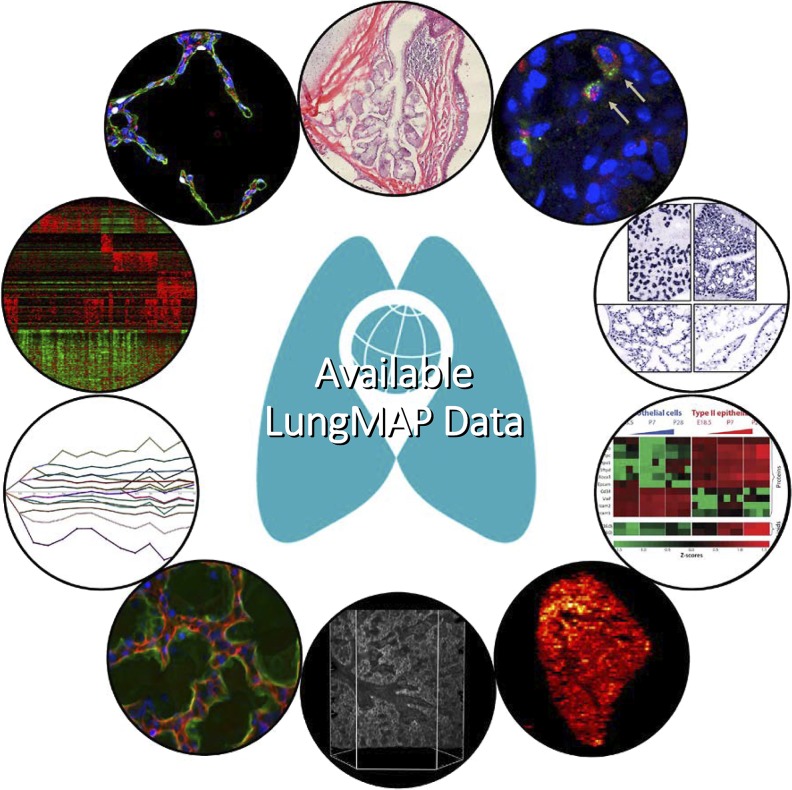
The LungMAP data. From *top*, clockwise: reference hematoxylin and eosin histology; multiplex fluorescent in situ hybridization with overlaying feature annotations; high-throughput in situ hybridization; proteomics and lipidomics; imaging mass spectrometry; Vibra-SIMM; 3-dimensional (3D) confocal imaging; profiling mRNA, miR, and methylation during development and maturation; single-cell RNA-seq and related methods; quantitative multicolor immunohistochemistry.

The LungMAP initiative had its origins from discussions at the National Heart, Lung, and Blood Institute (NHLBI) Workshops on Cell Plasticity in Lung Injury and Repair in April 2010 and on Molecular Determinants of Lung Development in September 2011. The NHLBI Board of External Experts organized the LungMAP initiative to build a molecular atlas of late-stage lung development to serve as a platform for discovery research to better understand critical events, including alveologenesis. This atlas would integrate multiscale information of gene expression, 3D cellular structures, and key morphogenesis events to provide detailed molecular anatomy of the developing mouse and human lungs. Figure 2 displays the scope of data types being generated by the Research Centers (RC). The database currently houses data from a variety of experiments across select stages of mouse development (canalicular through alveolar). Similar data in human lung is being generated across the RCs and is now being added to the BREATH database as we work to establish a comprehensive and extensive bank of mouse and human data.

The goals of the LungMAP initiative are to build *1*) a mouse lung atlas from embryonic day 16.5 to postnatal day 28 that integrates gene expression, imaging, and anatomic analysis, *2*) a human lung atlas to validate the molecular profile in lung cell types in normal human lungs from gestational week 23 to postnatal year 10, and *3*) an integrated, publicly accessible, and expandable database to accommodate existing and newly generated data.

**Fig. 3.**
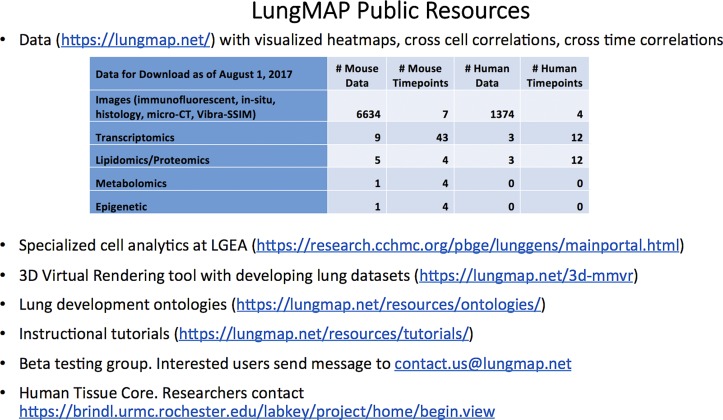
The LungMAP resources. LungMAP project resources include public data, specialized analytic tools at complementary consortium websites, educational resources, and opportunities for involvement.

The LungMAP consortium consists of a set of research centers, many of which represent multiple institutions with distinguishing expertise, engaged in collaborative activities to better understand the molecular processes that guide lung development, specifically during the stages of alveolar formation and development. This exploration is multipronged, including molecular (transcriptomics, single-cell transcriptomics, noncoding RNA, epigenomics, proteomics, lipidomics, and metabolomics), visual (confocal imaging, in situ hybridization, advanced chemical imaging, and 3D imaging), and computational (bioinformatics, systems biology) approaches. Research Centers are supported by a Human Lung Tissue Core and a Data Coordinating Center. The centers also participate in the sharing of resources such as mouse tissue and cells from a single well-controlled and characterized mouse colony operated by one of the LungMAP research centers. The resources currently supported by the LungMAP consortium that are available to the public are highlighted in Fig. 3.

The Cincinnati Children’s Hospital Medical Center (CCHMC) Research Center (RC) is responsible for single cell transcriptomics and confocal imaging and, as a member of the Data Coordinating Center (DCC), is developing computer programs for specific analyses. The RC generates and analyzes detailed NextGen gene expression data for human and mouse lung, including from single dissociated cells. Although many of the genes and networks regulating lung formation are shared among vertebrate species, the physiology, structure, extracellular matrix, region-specific cell types, and gene expression patterns vary between murine and human lung (17, 25). To effectively translate data from mouse models to humans, it is critical to understand their molecular similarities and differences at the cellular level. CCHMC is generating a detailed cell-specific RNA database for all human and mouse lung cell types of the lung parenchyma. Methodologies are being developed for single-cell and sorted-cell isolation, and computer programs for analysis of single-cell RNA, including Sincera (8), SLICE (7), LungGENS, LungIMAGE, and Lung Gene Expression Atlas (LGEA) (6), have been produced for the LungMAP project. Data and programs are available to all investigators. Single-cell analysis will be interpreted along with data derived from laser capture microdissection (LCM), fluorescence-activated cell sorting (FACS), and molecular imaging to create an expression map of the human and mouse lungs. CCHMC focuses on the analysis of cells from normal lung parenchyma (e.g., conducting airway and alveolar epithelial cells; vascular endothelial cells, including venous, arterial, lymphatic, and capillary cells; stromal and adventitial cells, including fibroblasts, pericytes, myofibroblasts, and cartilage; and neuroepithelial and neuronal cells). In addition, expanding on previous single-cell analysis on the lung by the CCHMC (5) and other investigators (22), the CCHMC will enhance the single-cell analysis of the lung with the application of Droplet sequencing (Drop-seq) to the single-cell analyses. This more recent technique will provide transcriptomic data for less abundant lung cell types, including lymphatic, mesothelial, and airway epithelial, as well as diverse cells of the hematopoietic and immune systems. At present, RNA sequences are being obtained from more than 5,000 individual cells from a single lung sample in each experiment. These data are being prepared for the LungMAP website at the present time. A new analytical pipeline based on Sincera (8) is being developed to handle the ever-expanding data being generated by Drop-seq.

CCHMC is also producing a library of lung images, including immunofluorescent confocal as well as hematoxylin- and eosin-stained sections from mouse and human samples. Acting as the Mouse Hub for LungMAP, CCHMC also provides mouse tissues to all research centers to reduce variability in murine-based research due to differences in substrain or environmental conditions.

The Pacific Northwest National Laboratory, Baylor College of Medicine, University of Washington, and Texas Advanced Computing Center together form a Research Center that applies high-throughput approaches to resolve the molecular changes of lung development specific to cell types and spatial location. This RC measures gene, protein, lipid, and metabolite levels in both mouse and human lung tissues. The multiomics characterization effort within this RC spans multiple scales from whole tissue to region-specific to cell-type specific analyses. State-of-the-art mass spectrometry (MS)-based proteomic, lipidomic, and metabolomic measurements are made on whole lung tissue samples, sorted cell populations (e.g., alveolar type II cells and mesenchymal, immune, and endothelial cells), and laser-capture microdissected (LCM) alveolar septae (3, 4). Spatial context in the mouse is provided by imaging the distribution of specific molecular entities across sets of thinly sliced lung tissue sections. Cell-specific semiquantitative gene expression measurements are acquired at submicron resolution using high-throughput in situ hybridization (HT-ISH) with digoxygenin-labeled probes for mRNA (1, 2). HT-ISH complements CCHMC RC’s RNA-Seq association of genes with cellular subtypes by providing spatial maps of the distribution of cells expressing genes. RNA distribution patterns are visualized at four time points in mouse [embryonic day 16.5 (E16.5), embryonic day 18.5 (E18.5), postnatal day 7 (P7), and postnatal day 28 (P28)] by HT-ISH for each gene, enabling quantitative comparisons across time and elucidating the relationship between molecular spatial localization and lung development. Hundreds of lipid species and unique metabolites are quantified via nanospray desorption ionization mass spectrometry (nano-DESI) across the lung at up to 10-μm resolution (12, 13, 21). Using this molecular knowledge base in mouse as a foundation, this RC will apply these same imaging and omic measurement methods to human tissue in the upcoming years of the LungMAP project.

The University of Alabama at Birmingham (UAB), Yale University, University of California-San Diego (UCSD), and Carnegie Mellon University (CMU) comprise a single research center focused on the systems biology of alveolar development. This center is generating a compendium of the dynamic and regional changes in DNA methylation and chromatin accessibility, microRNA, mRNA, and proteins that occur during alveolar formation to generate a dynamic temporal regulatory model of normal alveologenesis in both the mouse and human. Despite significant progress in understanding normal alveolar formation, a comprehensive understanding of the dynamic regulatory networks of this process is lacking. For example, although we are aware that the regulation of alveolar growth (beginning at approximately embryonic day 15 and ending at postnatal day 30 in the mouse) depends upon integration of numerous signals from multiple (e.g., Wnt, TGF, Hedgehog, Retinoid) pathways (15, 18, 20, 24), there is little understanding of how these pathways interact in the complex and rapidly varying spatial microenvironment of the developing lung. Similarly, we lack a detailed understanding of the hierarchy and temporal dynamics of transcriptional regulation during lung development, including the tightly regulated temporal changes in epigenetic regulation of DNA, expression of regulatory RNAs such as microRNAs, changes in transcription factors, or alternative splicing events. This group is identifying these epigenetic, RNA, and protein changes in samples from laser capture microdissection (LCM) of developing alveoli and sorted lung cells collected at tight intervals to allow detailed analysis. LCM is done at UAB, with samples distributed to Yale University for miRNA and mRNA analysis, UCSD for DNA methylation, and UAB for proteomics. Data integration and computational model development is conducted at CMU to identify regulatory “control points” in alveolar development (9).

The Saban Research Institute at Children’s Hospital of Los Angeles is the LungMAP 3D-imaging Research Center (RC), which is investigating alveolar development in unique ways that are leading to new insights (23). This RC has developed a set of approaches to generate data that will create a comprehensive high-resolution spatiotemporal map of the molecular, genetic, and cellular events in the developing lung across multiple stages of alveolar development scalable from lung alveolar anatomy to molecules. This group is using newly modified high-throughput multiplex ISH with novel “slice and dice” Vibra-SSIM confocal technology, μCT, μMRI, and Phase Contrast X-ray (PCX). Imaging is validated first in mouse and then in human tissue and correlated digitally with novel HT-ISH and fluorescence immunohistochemistry (IHC) data, focusing on epithelial, mesenchymal, neural, lymphatic, and vascular markers together with a fine map of matrix topology. Digital image-processing technology is being developed to meld digital multiscale images of alveolar structure with cell-autonomous gene expression with extracellular matrix protein configuration. A novel viewer called the 3D μCT/Microscopy Volume Renderer (MMVR), developed by the RC, is now offered to the scientific community on the LungMAP website that allows researchers to explore lung images in a three-dimensional space, with the capacity to view internal airway passages as well as structures within the lung interstitium. This tool employs direct volume rendering using ray casting, a true volumetric representation of the image data.

The University of Rochester Medical Center, in collaboration with Seattle Children’s Research Institute, operates the Human Tissue Core for LungMAP. The Human Tissue Core (HTC) identifies and manages tissue sources to meet the overall goal of LungMAP to build an open-access comprehensive molecular atlas of the late-stage developing lung. The HTC procures, processes, stores, and distributes normal late-gestation, neonatal, and early childhood human lung tissue and dissociated cells for the Research Centers, networking with the United Network for Organ Sharing and their partners. LungMAP studies the biology of normal lung development, which makes tissue health a critical concern. The HTC collects transplantation-quality tissues through the International Institute for the Advancement of Medicine (IIAM; http://www.iiam.org) and the National Disease Research Interchange (NDRI; http://ndriresource.org), organizations that link donors to the scientific community. A special effort, the Neonatal Organ Donation Program, was established by IIAM to provide donations to LungMAP and other important research efforts. IIAM and NDRI receive referrals to families wishing to donate for research when transplantation is not an option.

As part of HTC processing, donated lungs are imaged by computed tomography (CT) that can be reconstructed to provide 3D images and then processed for histological analysis or dissociated and sorted to provide a range of enriched cell populations, including subsets of epithelial, endothelial, mesenchymal, lymphatic, immune, and stem cells. A working group of highly qualified pathologists work with the HTC to establish biorepository and assessment tools and review specimens to assure quality and consistent selection of the tissues chosen for LungMAP.

The HTC maintains a laboratory information management system (LIMS) called BRINDL (https://brindl.urmc.rochester.edu) for tracking and identification of the derived products of the collection, including glass slides, paraffin and frozen blocks, cell fractions, RNA, digital CT and whole slide images, and associated donor data. The HTC LIMS system integrates with the LungMAP database for transfer of pertinent donor and tissue information. BRINDL provides complete specimen repository functions for tracking and managing requests to handle the traffic between the RCs and the HTC repository.

The Duke Clinical Research Institute and RTI International operate the Data Coordinating Center (DCC) for LungMAP, with added bioinformatics expertise from CCHMC. The DCC developed and maintains the LungMAP website and database called Bioinformatics REsource ATlas for the Healthy lung (BREATH). The DCC processes data submissions from the RCs, integrates data into BREATH, works with centers on data display, and coordinates with the HTC. The DCC also serves as the administrative infrastructure to facilitate collaboration across the consortium, creating consortium-wide priorities and policies, communication plans, and coordinating working groups and steering committee meetings. The DCC is also responsible for, and with LungMAP working groups has already developed, greatly expanded mouse- and human-specific lung ontologies for data annotation and integration and for tools to enhance sharing of LungMAP data with the scientific community and the public.

## BREATH: LungMAP Data Resource

With the exponential growth of omics data, a major challenge of LungMAP is the determination and development of an effective way to integrate and to offer to the community the multiscale information that links anatomic structure and cell types with epigenetic, RNA, protein, lipid, metabolic, signaling, and molecular and cellular interaction data.

Of fundamental importance to meet this challenge is the establishment of a database that can receive the complexity of data types generated by LungMAP and allow these data to be linked in myriad ways needed to investigate relationships. The DCC chose to build a triple-store database as the backbone of BREATH based on open-source OpenLink Virtuoso (16). Triple-store databases are optimized for storage and retrieval. Database structure consists of data “triples” built as subject-predicate-object. Triple-store databases are ideal for ontology-based applications and for linking disparate data types without requiring customized tables. Relations are established through linking, and a piece of data can be linked in myriad ways to support integration and comparison across groups, such as developmental time point, assay type, etc. BREATH data are interrogated through queries using SPARQL language (10).

**Fig. 4.**
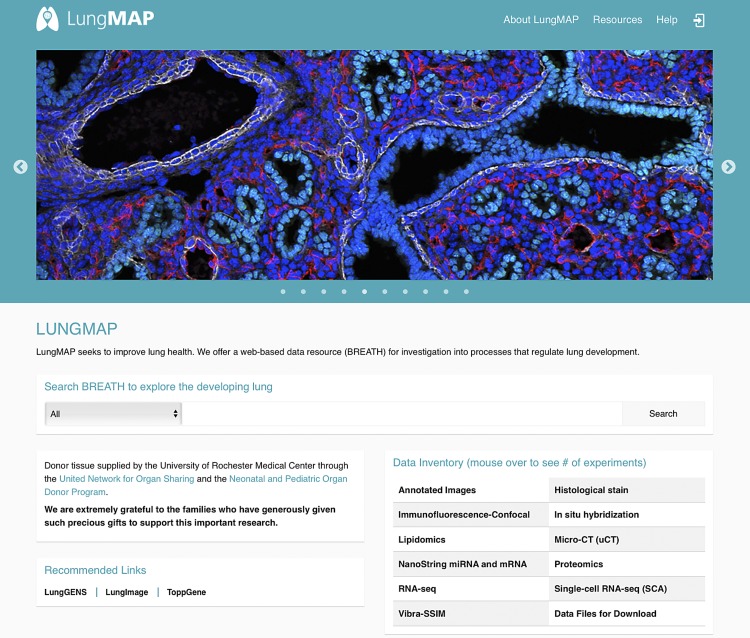
The LungMAP home page. Home page for www.lungmap.net, the official website for the LungMAP project.

A primary output to date for the DCC is the establishment and development of the LungMAP website (www.lungmap.net) (Fig. 4). The website consists of public facing pages, a private administrative and communication portal for LungMAP consortium members, and a freely accessible public database of lung development images and molecular data with tools to search and analyze them. Within the LungMAP website are links to other important websites of interest to the research community, including the site of our DCC partner CCHMC: Lung Gene Expression iN Single-cell (LungGENS) (5), Lung Gene Expression Atlas (LGEA), and Lung Image (LungIMAGE). The LungMAP website also includes resources such as tutorials, glossary, and forum as well as significant new components under development that are described below.

## LungMAP Anatomic Ontology

The LungMAP high-resolution anatomic ontologies describe the anatomic structures, tissues, and cells of the developing human and mouse lower respiratory tracts, including the trachea, bronchi, bronchioles, and alveolar parenchyma of the lung, as well as the pulmonary vascular, autonomic, and immune systems of the lung. The LungMAP ontology incorporates well-characterized structural and functional anatomic components, distinct histological tissue compartments, and generic and specific cell types. The ontology provides a coherent set of terms incorporated into an interactive, searchable, web-based atlas for the integration of anatomic, cellular, molecular, and imaging data. This ontology is organized along the proximal-distal axis of the lower respiratory tract, encompassing prenatal and postnatal stages of human and mouse lung development. On the LungMAP website (https://www.lungmap.net/resources/ontologies/), users can access the ontology through a special browser or download PDFs or Microsoft Word files containing the ontologies. These files include Human Alveolar Stage, Mouse E16.5 (early canicular stage), Mouse E18.5 [early saccular stage, Mouse P0–P03 (mid-to-late saccular stage], Mouse P04–P36 (alveolar stage), Cell Ontology for Mouse Lung Maturation, and Cell Ontology for Human Lung Maturation. These documents are flat file views providing the LungMAP identification numbers for each term within the current deployed ontology (Human Version 1.7, Mouse Version 2.9).

## Unique LungMAP Tools

Consortium efforts, led by the DCC, have focused on several specific components whose importance emerged as the project progressed. These include development of *1*) a web-based mechanism for image annotation, *2*) a web-based mechanism to create “theme” pages for display on LungMAP, and *3*) a viewer to present image data in 3D. These efforts are in prototype development as of this writing, with products available to the public through the LungMAP website in 2017.

### Image annotation.

**Fig. 5.**
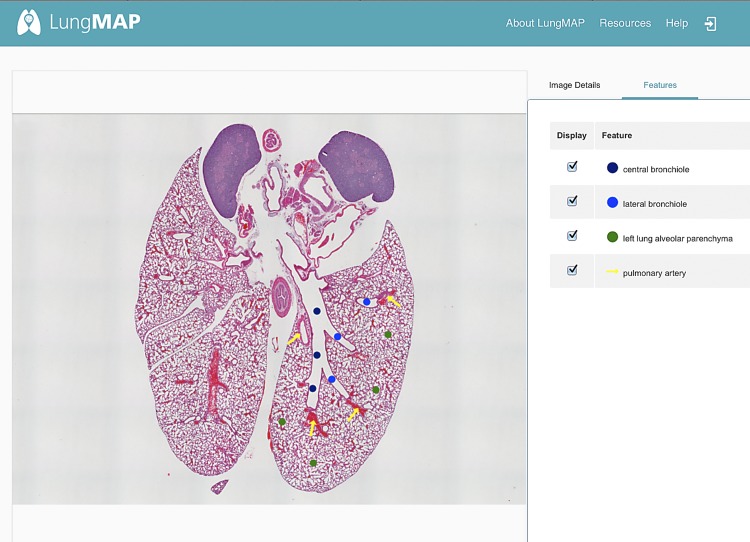
Annotated LungMAP image. Hematoxylin and eosin-stained whole lung from P07 C57BL6 male mouse. Annotated features are shown on the *right* side of the screen (*top* to *bottom*): central bronchiole, lateral bronchiole, left lung alveolar, parenchyma, and pulmonary artery. Multiple instances of these features are noted on the image.

Imaging data provide the foundation upon which to build an atlas of the developing lung. The LungMAP consortium has created a library of lung images using multiple imaging technologies that span from anatomic to molecular. To enrich understanding of these visual data, it was necessary to have a mechanism for identifying specific areas of an image to highlight the structures and labeling of the tissue, including proteins, cells, and larger anatomic features such as bronchioles and vessels that develop at certain time points. The LungMAP Image Annotation Tool allows a user to overlay images with feature symbols (dots, arrows, polygons), associated terms for structures, and notes. Annotators log into a secure site to access images in an environment that allows outlining of areas or placement of symbols onto the two-dimensional (2D) image with linkage to a term in the ontology. The tool is engaged by selecting any single image thumbnail from within an image experiment. Annotators manually place the symbol on the 2D image and add the additional information. The association of a symbol with the image (with descriptive information) is referred to as image annotation, as shown in Fig. 5. All image annotations are associated with the standardized terms in the LungMAP ontology, which enables linking of data across the website through the assigned term. Annotation completed with this web-based tool is a manual process conducted by lung development curators at the LungMAP RCs.

**Fig. 6.**
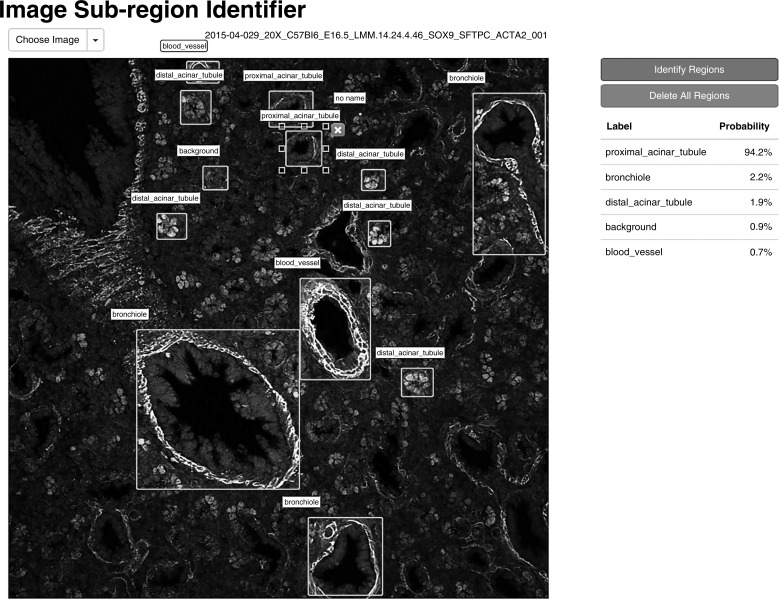
Automated LungMAP image annotation. Screenshot of interactive web application for automated identification of anatomic structures within bounding boxes drawn by user. Multiple regions of interest can be identified simultaneously. *Right*: posterior probability for each label for currently selected bounding box.

A second approach to image annotation that LungMAP is developing uses machine-based algorithms that automatically identify specific components of the image. The development team has assembled a database of characteristics for 4,557 confocal immunofluorescent image files. These metadata are being used to group collections of images (e.g., images from E16.5 mouse at ×20 displaying Hopx and Nkx2-1 using defined probes). They have successfully identified specific features of certain lung images by combining information from the metadata and image analysis routines from the OpenCV package (Python bindings; Fig. 6). This machine-learning approach is in its early stages, slated for further development and public display in 2017.

### StoryBuilder.

LungMAP has a twofold mission of executing research on normal lung development and broadly sharing and presenting results to audiences of varying scientific expertise. The challenge was to develop an easy-to-use tool that allows LungMAP researchers to present information to the public. This information can range from education on experimental procedures to cross analyses of LungMAP data for insights into the regulation of lung development. The tool required certain basic components to build a narrative format. A prototype of the tool (called StoryBuilder) was released for testing in late 2016, allowing researchers to build simple web pages within the LungMAP website. Ease of use was key to the design, with flexibility to incorporate text, image, and media to support presentation. Although in an early stage of design, plans for the next iteration include development of methods to incorporate LungMAP data directly into a constructed page for compilation and comparison of the many types of image and omics data. StoryBuilder will support consortium research as observations unfold and will provide a flexible forum for sharing as these conclusions evolve or change. StoryBuilder will allow researchers to support their observations with images, media links, and references to published literature (with proper acknowledgments/citations). Once made public on the LungMAP website, website viewers will have the opportunity to ask questions and post comments so that narratives are not static but can evolve as new research uncovers new discoveries.

## 3D MMVR

LungMAP offers a tool to visualize microscopy image data in a very unique way. An integrated input-output visualization tool called 3D MMVR (μCT-Microscopy Volume Renderer) does direct volume rendering using ray casting, a true volumetric representation of the data. Ray casting preserves detail and allows for the quick adjustment of opacities through controls in the user interface. Through the 3D MMMVR, a user can look inside lung structures to see internal airway passages, including cells contained within them. The 3D viewer opens a data set for the user to explore. Currently, the data are presented for adult mouse lung. All data are in a DICOM file structure. LungMAP will expand the use of this tool this year (2017) with more data and eventually invite outside researchers to use the tool with their own data to conduct analyses.

## Upcoming

LungMAP will continue to develop its lung data resource, adding additional data for both mouse and human samples. Expanded protein, lipid, and metabolomics data and single-cell expression and methylation data for additional key developmental time points are now being prepared for viewing and download. Models based on regulatory pathway analysis of lung development will be integrated into the website during this year (2017). Molecular and imaging data from lung researchers outside the LungMAP consortium and with databases such as Mouse Genome Informatics will be considered for downloads and integration with the BREATH database in 2018.

In the coming year, we plan to increase engagement of the community through direct outreach. We will expand our beta tester group to give feedback on web functionality, offer seminar sessions to explore the data with LungMAP researchers, and further develop LungMAP for educational purposes through efforts such as enhancing our 3D renderer for wider public engagement. Bringing students and tech developers into the LungMAP community will ensure that LungMAP is relevant to a broad and diverse base of users for the present and years to come.

## GRANTS

The work was supported by the National Heart, Lung, and Blood Institute Grants U01-HL-122638 (to M. E. Ardini-Poleske, Robert F. Clark, and S. M. Palmer), U01-HL-122642 (to S. S. Potter and J. A. Whitsett), U01-HL-122703 (to C. Ansong, J. P. Carson, and R. A. Corley), U01HL122700 (to G. H. Deutsch, T. J. Mariani, and G. S. Pryhuber), U01-HL-122626 (to N. Ambalavanan, J. S. Hagood, and N. Kaminski), and U01-HL-122681 (to D. Warburton).

## DISCLOSURES

No conflicts of interest, financial or otherwise, are declared by the authors.

## AUTHOR CONTRIBUTIONS

M.E.A.-P. and J.P.C. prepared figures; M.E.A.-P., R.F.C., and N.A. drafted manuscript; M.E.A.-P., R.F.C., C.A., J.P.C., J.S.H., G.S.P., J.A.W., and N.A. edited and revised manuscript; M.E.A.-P., R.F.C., C.A., J.P.C., R.A.C., G.H.D., J.S.H., N.K., T.J.M., S.S.P., G.S.P., D.W., J.A.W., S.M.P., N.A., and t.L.C. approved final version of manuscript.
